# Competence-based teaching and learning in the outpatient clinic: development of a clinical elective in ambulatory medicine

**DOI:** 10.3205/zma001244

**Published:** 2019-08-15

**Authors:** Vanessa Schimbeno, Catherin Bosle, Anka Stegmeier-Petroianu, Nima Etminan, Kristina Hoffmann

**Affiliations:** 1 University of Heidelberg, Medical Faculty Mannheim, Mannheim Institute of Public Health, Social and Preventive Medicine, Mannheim, Germany; 2 University of Heidelberg, University Hospital Mannheim, Department of Neurosurgery, Mannheim, Germany

**Keywords:** curriculum development, outpatient clinics, ambulatory medicine, competency-based education, interest-based learning

## Abstract

**Aim:** Although physicians have the most contact with patients in the outpatient setting, topics relating to ambulatory medicine have been less present in medical education. To teach professional skills, practical learning opportunities must be created in which students can practice medical skills under authentic working conditions in the outpatient setting. The clinical elective in ambulatory medicine has been developed, evaluated and revised at the Mannheim Medical School as a practical, competency-based learning experience in outpatient clinics (liver clinic, psychiatric outpatient clinic, uro-oncological consultations, etc.).

**Methods: **The elective was designed using the plan-do-check-act (PDCA) cycle in the form of a longitudinal practical course during the fourth year of study. By selecting one of four concentrations in ambulatory care, students have the opportunity to focus on and pursue an individual interest. Students are given assignments during three separate sessions at an outpatient clinic and complete a log book which contains the learning objectives, assignments and grading criteria. Once the elective had been completed, the students (n=165) and mentors (n=7) had the option to participate in a survey to evaluate knowledge gain and satisfaction with the elective.

**Results: **The students rated their personal growth in knowledge about common diseases and patient-centered communication positively, while rating their knowledge gain in ambulatory patient management somewhat lower. The first offering of the elective was evaluated as satisfactory by students and mentors. In 85.8% of the cases, students reported that they would recommend the selected outpatient clinic for this course. Suggestions for improvement, such as those concerning the log book and scheduling system, were considered during the first revision.

**Conclusion:** This elective provides an opportunity to become familiar with ambulatory medicine in a practical and competency-based manner during medical studies. Skills in ambulatory medicine are already taught and applied in the fourth year of study in a practical setting and also deepened further through interconnections with other courses and chosen concentrations. Moreover, this elective format may be used by other medical schools depending on which aspects of ambulatory medicine are focused on.

## Zusammenfassung

**Zielsetzung:** Obwohl im ambulanten Bereich der häufigste Patientenkontakt zu Ärzten stattfindet, sind im Medizinstudium ambulante Inhalte bislang wenig repräsentiert. Zur Ausbildung beruflicher Handlungskompetenz müssen praxisorientierte Lerngelegenheiten geschaffen werden, in welchen die Studierenden medizinische Fertigkeiten unter realistischen Arbeitsbedingungen im ambulanten Setting üben können. An der medizinischen Fakultät Mannheim wurde dafür das klinische Wahlfach *Ambulante Medizin* als praxis- und kompetenzorientierte Lerngelegenheit in Klinikambulanzen (z.B. Leberambulanz, Psychiatrische Ambulanz, uro-onkologische Sprechstunde) entwickelt, evaluiert und überarbeitet.

**Methoden: **Das Wahlfach wurde unter Verwendung des Plan-Do-Check-Act (PDCA)-Zyklus als longitudinale Praxisveranstaltung im 4. Studienjahr konzipiert. Dabei wird durch die Wahl eines fachlichen Schwerpunktbereichs eine Möglichkeit zur individuellen Neigungsorientierung geschaffen. Studierende übernehmen in drei Terminen Aufgaben innerhalb der Klinikambulanzen und führen ein Logbuch, in dem Lernziele, Aufgaben, sowie Bewertungsschema dokumentiert sind. Am Ende des Wahlfachs konnten Studierende (n=165) und Betreuer (n=7) an einer Befragung zum Lernzuwachs sowie der Zufriedenheit mit dem Wahlfach teilnehmen.

**Ergebnisse:** Studierende bewerteten ihren individuellen Lernzuwachs zu häufigen Krankheitsbildern und patientenzentrierter Kommunikation hoch, während dieser im ambulantem Patientenmanagement etwas geringer bewertet wurde. Der erste Durchlauf des Wahlfachs wurde von Studierenden und Betreuern als befriedigend bewertet. In 85,8% der Fälle gaben Studierende an, dass sie die gewählte Ambulanz für die jeweilige Lerneinheit weiterempfehlen würden. Verbesserungsvorschläge, wie die Ausgestaltung des Logbuchs oder des Anmeldesystems, wurden in der ersten Revision berücksichtigt.

**Schlussfolgerung: **Das Wahlfach bietet eine Möglichkeit, bereits im Studium ambulante Medizin praxis- und kompetenzorientiert zu erlernen. Kompetenzen für die Ambulanz werden bereits im vierten Studienjahr in einem praxisnahen Setting erlernt und angewendet, sowie durch die Verknüpfung mit anderen Veranstaltungen und die individuelle Schwerpunktsetzung besser vertieft. Weiterhin ist die Übertragung des Wahlfachs unter Berücksichtigung unterschiedlicher ambulanter Schwerpunkte auf andere medizinische Fakultäten möglich.

## 1. Introduction

German law mandates the assurance of adequate healthcare for the people [https://www.gesetze-im-internet.de/sgb_5/BJNR024820988.html]. This has special significance for ambulatory care since this is the setting where most interactions between patients and physicians occur [1]. In addition to care provided by doctors in private practice, ambulatory care is increasingly taking place in outpatient clinics, a change that has been supported by the Federal Joint Committee (Gemeinsamer Bundesausschuss) and encouraged by various amendments to the statutes, such as the German Social Code and the acts to modernize statutory healthcare and strengthen competition among statutory healthcare insurers and providers [2], [3].

However, these developments are still not given sufficient consideration in current medical education since during medical study the contact between doctors and patients occurs primarily in the hospital inpatient setting [4]. As a result, opportunities to learn about common clinical pictures and courses in the ambulatory setting are much more seldom [4]. In respect to medical expertise and decision making, students feel underprepared for primary care due to a lack of knowledge regarding the organization and provision of outpatient care [5]. The ability to make appropriate decisions in different professional situations is, however, essential to a fully rounded and comprehensive education [6].

Traditionally, the places where outpatient medicine is mainly taught in German medical degree programs are primary care practices, where the block practicum in general practice and four-week-long clerkships are served, and as such are firmly associated with the field of general practice. However, depending on the medical school, general practice as a subject can have a greater or lesser presence in the curriculum and thus affect the amount of teaching dedicated to ambulatory medicine.

The expansion of education in the context of ambulatory medicine is being realized in different ways by German medical schools. While some medical schools are expanding training in ambulatory medicine within the general practice curriculum and at private primary care practices [7], [8], [9], others, such as the model curriculum in Jena, are offering an “elective thread” that covers topics pertaining to ambulatory medicine in multiple subjects [10].

In the MaReCuM model curriculum at the Mannheim Medical School of the University of Heidelberg [https://www.umm.uni-heidelberg.de/studium/modellstudiengang-medizin/], topics in ambulatory medicine and related learning opportunities are offered mainly in the fifth year of study within the scope of general practice (e.g. the two-week-long block practicum in general practice). An academic quarter dedicated to ambulatory medicine was already established in 2011 when the final practical year of medical study was divided into four rotations. Instruction during this quarter takes place at university outpatient clinics, specialty physician practices and primary care practices [11] in an effort to expand education to include learning in other ambulatory settings. In the course of developing the ambulatory medicine curriculum further, learning opportunities are used starting in the third year of medical study to develop a coherent, longitudinal module in ambulatory medicine (LAMM=**L**ongitudinal module **A**mbulatory **M**edicine **M**annheim).

The format of the fourth-year elective in ambulatory medicine was implemented within this framework and is structured the same as the ambulatory care rotation during the final year in that it focusses on the following four concentrations: conservative/chronic medicine, interventional/surgical medicine, oncology, psychiatry and psychotherapy (see comments in table 1 (Tab. 1)). The aim was to also include the outpatient clinics at the hospital as a practical professional learning opportunity with experienced MaReCuM instructors during an earlier study phase in order to foster and encourage continual development of competency in providing outpatient care.

In the following we describe the development, evaluation and revision of the competency-based clinical elective in ambulatory medicine. In keeping with the elective’s development, we then discuss four educational criteria which the elective needs to meet:

1. early exposure to ambulatory medicine and the related responsibilities,
2. utilization of practice-based learning in an authentic professional setting,
3. synthesis of previously learned knowledge and skills from prior courses in the outpatient setting, and
4. the option to pursue a special focus in the field of ambulatory medicine.

Finally, the possibility to implement such an elective at other German medical schools is explored.

## 2. Project description: The clinical elective in ambulatory medicine

The PDCA cycle (plan-do-check-act; [9]) provided the organizational framework for curriculum development. The advantage of this cycle lies in its adaptability to different content, the possibility to apply ongoing quality improvements, and the simplified communication with staff who are not involved in curricular development [9]. The following five steps were taken:

1. **P**LAN: In the **planning and development phase**, after setting the main goals in alignment with the university’s institutional structures, the learning objectives, curricular content and teaching formats must then be defined and coherently formulated for implementation along a specific timeline.
2. **D**O: The **implementation phase** for the newly developed curriculum begins with informing and communicating with all key actors. Implementation is continually monitored and viewed in terms of the entire medical curriculum.
3. **C**HECK: After the initial implementation comes internal quality assurance in the form of an **evaluation** to verify the meeting of goals and analyze the potential for improvement.
4. **A**CT: Based on which potential improvements have been identified, the newly developed curriculum then undergoes a **revision phase** in which it is adjusted accordingly and continued in the revised form.

### PLAN: Design and development

The development of the elective began as part of the modifications to the study program in the 2016 summer semester with the appointment of a responsible coordinator. Members of the LAMM project and the Office of the Dean of Studies were involved in the entire developmental process. In line with the recommendations by Schaper [12] and other stakeholders in medical education were also included from the start in the development of the new elective in ambulatory medicine. The elective was implemented in the fourth year of study. Analogous to the ambulatory care rotation during the final year, students were able to select a concentration and register for three dates, or sessions, at selected university hospital outpatient clinics (see table 1 (Tab. 1)). The students were free to determine where the three sessions would be spent without being limited to one single outpatient clinic. Overall, 56 different outpatient clinics at 18 hospitals belonging to the Mannheim Medical School stood ready to participate in four possible concentrations. Private medical practices have not been included initially due to capacity issues because of their presence in other courses, such as the block practicum in general practice.

The current length of the elective was set at a minimum of 15 hours, divided into three scheduled sessions to accommodate the different consulting hours at each clinic. The criteria for passing the elective were defined as attending all three sessions, completing all log book assignments (see below), and submitting an evaluation of the student’s performance by the particular mentor in the outpatient setting.

During each session spent in the outpatient setting, subject material specific to ambulatory medicine and the related learning objectives were to be covered (see table 1 (Tab. 1)). The learning objectives were based on the definitions in the National Competency-based Catalogue of Learning Objectives for Undergraduate Medicine (NKLM) [13] but were, nonetheless, adapted to the practical instruction in this ambulatory setting. A log book with learning objectives, assignments and grading criteria was created for students and their mentors to serve as documentation and guidance. Students were thus better able to measure their own progress, which in turn assisted in the learning and practicing of relevant skills [14]. The requirement to carry out defined tasks during each session ensures a uniform level of practice-based teaching and learning. As a consequence, it also assures that the times scheduled for the elective were used to acquire competencies and not spent simply observing.

An online scheduling system was developed for entering and administering the times to be spent by the students in the outpatient setting. The learning management system Moodle [https://moodle.org/], well-known and often used by the Mannheim Medical School, served as the virtual platform for this. The educational concept was then presented to the academic studies committees belonging to the Office of the Dean of Studies for quality assurance. Since representatives from the outpatient clinics and medical student sit on these committees, it was possible to gather expert opinions on the concept and use them in the course of further development.

#### DO: Pilot 2016/2017

##### Implementation of the elective

The elective in ambulatory medicine was first implemented in the 2016/17 academic year. At the beginning of the winter semester the responsible coordinator presented the elective’s content and procedure to fourth-year students and mentoring physicians. In addition, the participating outpatient clinics received a procedural manual for the elective, a sample log book and instructions for the scheduling system. Participating were primarily outpatient clinics whose medical staff already taught MaReCuM courses so that medical teaching experience had already been gathered. Students were able to sign up for clinics in their area of concentration using the scheduling system (see table 1 (Tab. 1)). After a brief introduction and meeting with the mentoring physician, the students then accompanied the outpatient clinic staff during each patient appointment for at least five hours. During this time, the students were required to do one or more assignments in the log book and present their work to the mentor (see table 1 (Tab. 1)). At the end of each session the students received feedback and a written evaluation from the mentor regarding the assignments, their communication skills and attitudes toward patients and clinic staff, as well as on the techniques used to compile case histories and conduct physical examinations.

##### Methods of Evaluation

To evaluate the aims of the elective, students were surveyed regarding their subjective individual growth in knowledge and satisfaction with the practice-based teaching at the outpatient clinics. Also, students rated their satisfaction with the range of choice of outpatient clinics, the scheduling system, the sessions, and use of the log book as a learning tool. Each item was given a five-point Likert scale ranging from 1 (very good/high) to 5 (inadequate/low) and students could make suggestions for improvement in the form of open-ended written texts. Quantitative analyses were scored conventional German academic grading scale (e.g. 1.5 to 2.4 = good), while the open-ended responses were analyzed for frequency of similar suggestions. Finally, the overall satisfaction with the elective was assessed. The mentors at the outpatient clinics received an equivalent online survey at the end of the fourth study year for the purpose of evaluating the elective. The evaluations were carried out in conformance with the University of Heidelberg’s regulations.

## 3. Results

### CHECK: Evaluation results

A total of 56 outpatient clinics and 193 students participated in the elective in ambulatory medicine, of which 165 (85.5%) students completed the evaluation survey. Seven instructors also participated in the evaluation.

Students’ subjective growth in knowledge regarding the clinical pictures common to the different outpatient settings (*M*=1.9; *SD*=0.9) and patient-centered communication (*M*=2.2; *SD*=1.1) received the highest ratings. The knowledge gain regarding the possibilities and limits of outpatient treatment (*M*=2.4; *SD*=1.1) and the systematic recording and evaluation of the course of a disease (*M*=2.4; *SD*=1.2) was also rated positively. The students rated their subjective knowledge gain regarding ambulatory patient management (*M*=2.7; *SD*=1.2), processes and responsibilities in outpatient clinics (*M*=2.7; *SD*=1.2), and contacts and connections with other medical institutions (*M*=2.6; *SD*=1.2) as “satisfactory.”

**Figure 1:**
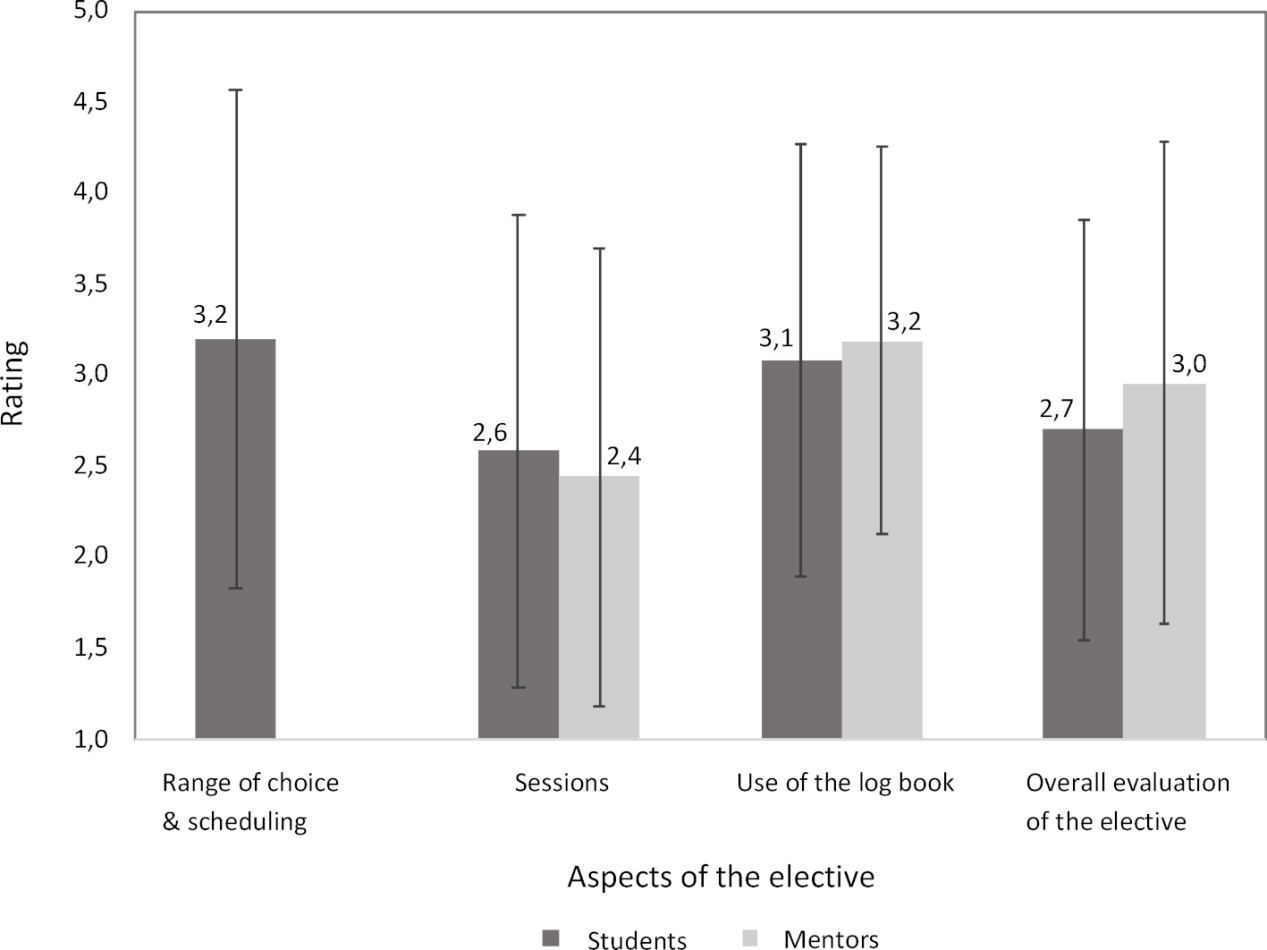
Mean values for the evaluation regarding the satisfaction of students (N=165) and mentors (N=7) with aspects of the elective in ambulatory medicine during the 2016/17 academic year. This evaluation used a five-point Likert scale (1=very good; 5=inadequate).

The results of the evaluation regarding the satisfaction of students and mentors are presented in figure 1 (Fig. 1). On average, those surveyed rated the elective as overall satisfactory (students: *M*=2.7: *SD*=1.1; mentors: *M*=3.0; *SD*=1.4). The analysis of the open-ended responses yielded suggestions from students and mentors for revising the log book as a way to improve the elective. Students expressed the wish for better alignment between the assignments in the log book and the tasks assigned in the individual clinics. The surveyed mentors confirmed that using the log book was difficult given the extensive content and the high workload in the clinics and that a coherent sequencing of the three scheduled sessions at the clinics was not visibly reflected in the log book. In addition, many of the outpatient clinics were too specialized to meet the learning objectives in the log books or to enable the students an adequate opportunity to actively participate. Moreover, the suggestion was made to more regularly update the entries in the scheduling system. Positive comments were made regarding the motivated mentorship in the outpatient clinics and the professional interaction with the staff. Students found patient recordkeeping and drafting doctor’s reports to be helpful learning opportunities. Students described the elective as giving them interesting and practical insights into ambulatory care. In addition to the assignments in the log book (see table 1 (Tab. 1)), students could conduct in part independent patient consultations and examinations. In 85.8% of the cases, students indicated that they would recommend the chosen outpatient clinic to others for this elective.

#### ACT: Module revision

As part of quality assurance, the developers, the mentoring physicians and the fourth-year student representatives revised the elective in August 2017. The revised program started in the 2017/18 winter semester. The following changes were made to the elective based on the discussion of the evaluation results and the reports on practical implementation of the elective:

1. **Outpatient clinic descriptions:** The hospitals were encouraged to list information about their outpatient clinics on the scheduling system. This included descriptions of organizational details (office hours, contact people), the range of medical services and the most important treatments offered by the hospital’s outpatient clinics. Based on this information, students are able to choose a hospital where they can learn or hone skills that match their interests.
2. **Specific subjects within a concentration:** To ensure medical and curricular coherency, students select a hospital at which they wish to take the elective after they have chosen a concentration. The students are free to choose whether they spend the sessions at the same outpatient clinic or at different outpatient clinics in the chosen hospital. Prior to scheduling specific sessions, an overview is provided of the hospitals and their particular specialties.
3. **Log book: **The learning topics for each elective session in the outpatient setting were revised so that they better represent working conditions and the practical learning opportunities for the students while at the outpatient clinics (see table 1 (Tab. 1)). Main learning objectives for each session are predefined, and learning objectives specific to the outpatient clinics are identified by the mentors during each session. The main learning objectives form an educational framework [15] in which the medical and organizational aspects connected with each outpatient clinic are reflected in the formulation of specific learning objectives. The focus of the assignments is on patient management, clinical examination, and oral and written case presentation. These are addressed during the outpatient consultations in order to enable independent and meaningful work for students during all sessions. Evaluations and final feedback are given during the final session. The evaluation criteria have been altered to match the new assignments and reflect the expectations for excellent and inadequate performance (see “rubric-approach” [16]) in order to give the mentors uniform rubrics for the evaluations.

## 4. Discussion

Although the most frequent contact between doctors and patients is seen in the outpatient setting, there are only very few opportunities for practical, competency-based learning in ambulatory medicine and these curricular units are often found relatively late in medical curriculum [1], [4], [17]. Against this background, the elective in ambulatory medicine was implemented, evaluated and revised as part of the MaReCuM study program offered by the Mannheim Medical School at the University of Heidelberg. The competency-based nature of this elective is discussed in the light of the teaching criteria we defined for this elective along with its suitability for use at other medical schools.

Prior to implementing the elective in ambulatory medicine as part of the MaReCuM model curriculum, content pertaining to ambulatory medicine was presented in the early study phases in a mainly implicit and mostly theoretical manner. In terms of competency-based teaching and learning, this elective is meant to give students an opportunity to gather their first practical experience at an outpatient clinic in the fourth year of medical study—two years before the final practical year.

With its practice-based design, the MaReCuM model curriculum responds not only to educational mandates from lawmakers, but also to the many desires expressed by students to have more opportunity while in medical school to practice under supervision in professional settings [5], [18]. By assuming responsibility for tasks in a specific outpatient setting, students are placed in a context where they are able to apply and hone previously acquired theoretical knowledge and skills. In addition, students’ medical competencies specific to providing outpatient care are fostered in an authentic practical setting where there is, for instance, limited time for diagnoses and a need for professional communication with other healthcare institutions. The results of the first evaluation show that students rated their subjective gain in knowledge about common diseases and patient-centered communication very positively. In regard to topics such as processes and scopes of responsibility in the outpatient setting or connections to medical institutions providing further care, students reported a lower subjective gain in knowledge. The reason for this could have been the length of 15 hours stipulated for the elective, since it was too brief to establish familiarity with such detailed processes in addition to normal patient contact. Competency-based learning requires active, solution-based engagement with the subject material [19]. Students found the practice-based – and thus active – learning opportunities, such as patient recordkeeping and drafting doctor’s reports, to be very positive. Working together with an experienced outpatient physician, students were given direct feedback on their performance and were able to realistically assess their own level of competency [20].

This elective offers more than the chance to build on earlier medical coursework: it also links to later (ambulatory) coursework, specifically the ambulatory quarter in the final year of study (see below). Through repetition, the students attain a higher level of professional competency in ambulatory medicine and become truly prepared for the demands of professional practice through the synthesis of particular areas of knowledge and skills [12]. The MaReCuM model curriculum thus follows the recommendation to practically impart interdisciplinary skills and competencies, such as the recognition and treatment of typical diseases, over the entire course of study [21].

Analogous to the final-year quarter dedicated to ambulatory medicine, this elective focuses on aspects of medicine that can be pursued depending on an individual student’s interests. This makes it possible in the fourth year of study to concentrate on an individual subject that will be covered again during the final year, as befits a longitudinal curriculum [22]. The opportunity to select a subject specialty based on personal interest can promote students’ motivation to learn, something that should lead to qualitatively better learning outcomes [19]. Students learn material that they find interesting more independently and use more challenging learning strategies to deepen their knowledge of the learned material. This contributes not only to the perception of being competent, but also to better learning [19]. Hence, we recommend offering the option to select a concentration or even a specific subject as part of the curriculum. [19], [23].

Generally, the elective is rated positively by students for its relevancy to practice, and students recommend the opportunity to gather experience in a practical setting to others.

### The elective’s suitability for use by other medical schools

At some medical schools in Germany curricular changes are already being made to improve teaching and learning in ambulatory medicine. This encompasses the implementation of learning opportunities dedicated solely to general practice in early study phases [24], [25], mentor programs ranging from the “GP track” [8] to comprehensive medical education focused on ambulatory medicine across all subjects in the form of an ambulatory-based curriculum (AOM; [10]).

The clinical elective in ambulatory medicine implemeted by the Mannheim Medical School is an interdisciplinary option to expand the medical curriculum by offering more contents in ambulatory medicine. It is possible to design and implement equivalent courses in other conventional or model medical curricula due to the elective’s organization and the way in which the content is structured. This elective does not replace any existing courses, but is rather a supplemental course on outpatient care. It can be used to teach medical competencies for both inpatient and outpatient settings [4].

The main learning objectives for ambulatory medicine and the assignments allow the elective to cover different medical contexts. Defining a range of concentrations enables school-specific design and prioritization in the selection of subject areas. As a result, specific student profiles and academic interests can be defined as special features [23]. Links to content across the curriculum to existing theoretical courses or practical learning units in the inpatient setting can also be implemented in which students can continually use and expand their medical expertise and skills in different professional settings. In addition, general skills and competencies – essential for medical education and professional qualification – are reflected in the main learning objectives [15].

The flexible scheduling of the three sessions for the students and the outpatient clinics allows incorporation into the clinical phase of study with little effort. The use of the medical school’s learning management system facilitates easy scheduling.

By using hospital outpatient clinics as practical learning settings, existing structures at specific medical schools can be integrated easily. With regular inclusion of outpatient learning opportunities in the curriculum, teaching and learning at German medical schools can indeed meet international standards [26]. By spreading students out over many hospital outpatient clinics, large students cohorts could even take and complete this elective, making implementation as a required course also conceivable.

#### Limitations

The pilottesting and its evaluation show some challenges in the development of learning opportunities in ambulatory medicine which we would like to address here.

The rather specialized university clinics are very different from each other regarding the spectrum of diseases and patients and do not represent the typical range of diseases seen in primary care. Still, there were some common features of the ambulatory work environment, such as a rapid sequence of patients, time pressure [27], and the identification of indications based on outside findings of differing quality [4], all of which give students valuable experience in terms of outpatient care.

To ensure appropriate learning of ambulatory skills and competencies, the plan it to focus the elective on outpatient clinics with a wider spectrum of patients and treatments and to include highly specialized clinics only during the ambulatory quarter in the final year. Parallel to teaching in the hospital outpatient settings, the courses in primary care offered at Mannheim Medical School will be expanded to go beyond the traditional clerkships, block practicums and final practical year and will be synthesized into an innovative educational concept in ambulatory medicine.

The main learning objectives that were articulated during the developmental phase were not always fully met by the specifics of certain outpatient clinics in that not all learning objectives could be adequately covered during the students’ time on site. Through the addition of learning objectives specific to ambulatory medicine, the elective objectives are formulated as differentiated sub-competencies, a change that is meant to increase specificity in the various outpatient clinics.

The coordination of a large number of outpatient clinics and teachers posed a challenge in terms of communication between the Office of the Dean of Studies, the mentors and the students. The informational materials were well received by the mentors and perceived as helpful in planning the time spent by the students at their clinics. With a total of 56 outpatient clinics covering the four concentration, it was not possible, when designing the elective, to take individual aspects into consideration in terms of range of treatment, diagnostics or patient clientele. As a consequence, both students and mentors questioned the suitability of some outpatient clinics as a learning environment for a fourth-year elective. For this reason, mentors from all specialty areas were invited to participate in the module’s revision, and an additional evaluation of the elective was designed for the mentors.

The elective evaluations measured student and mentor satisfaction and the students’ subjective gain in knowledge. In addition to these, an objective measure of knowledge gain would be meaningful for quality assurance. For this reason, an ongoing process and outcome evaluation is to be established, for which the PDCA cycle has proven itself to be an easily understandable and practical method for quality improvement in practice-based teaching and learning.

## 5. Conclusions

The elective in ambulatory medicine was implemented in the MaReCuM model curriculum meeting predefined educational criteria. Overall, the first implementation was evaluated as satisfactory by students and mentors. Even if measures to improve quality in education have already been taken, ongoing evaluation and development of this elective are important. The previously implemented PDCA cycle will continue to be used for this. The intention is to expand the elective for future cohorts to include additional learning units such as e-learning and theoretical instruction. In addition, general practitioners, specialist physicians in private practice and municipal healthcare institutions (e.g. City of Mannheim’s Child & Adolescent Health Services) will be successively included as options for students to select. This will counteract the uneven selection of specialty areas and allow the elective to represent many more areas of ambulatory medicine [28]. Since this was the only elective at the Mannheim Medical School at the time of the evaluation, attention must be paid in coming years to how student numbers develop if other non-ambulatory electives are introduced.

## Acknowledgements

We wish to thank the following colleagues in the Office of the Dean of Studies at the Mannheim Medical School: Christine Gäbel and Lucia Trauner for schedule coordination and organization of the elective; the evaluation coordinator, Ana Bordes, for valuable cooperation; Dr. Katrin Schüttpelz-Brauns for critical comments and constructive feedback on the manuscript. We extend our gratitude to Dr. Elisabeth Narciss and Prof. Udo Obertacke (Competency Center PJ) for the valuable information and extensive information about the final-year quarter dedicated to ambulatory medicine. In addition, we thank Julia Liebnau (MIPH) for her help with literature research and creating the tables and figures. A special thanks goes to David Litaker, MD, Ph.D. for his advice and guidance during the writing process.

## Funding

Funding was received from the Ministerium für Wissenschaft, Forschung und Kunst Baden-Württemberg (MWK), number 42-04HV.MED(17)/8/2.

## Competing interests

The authors declare that they have no competing interests. 

## Figures and Tables

**Table 1:**
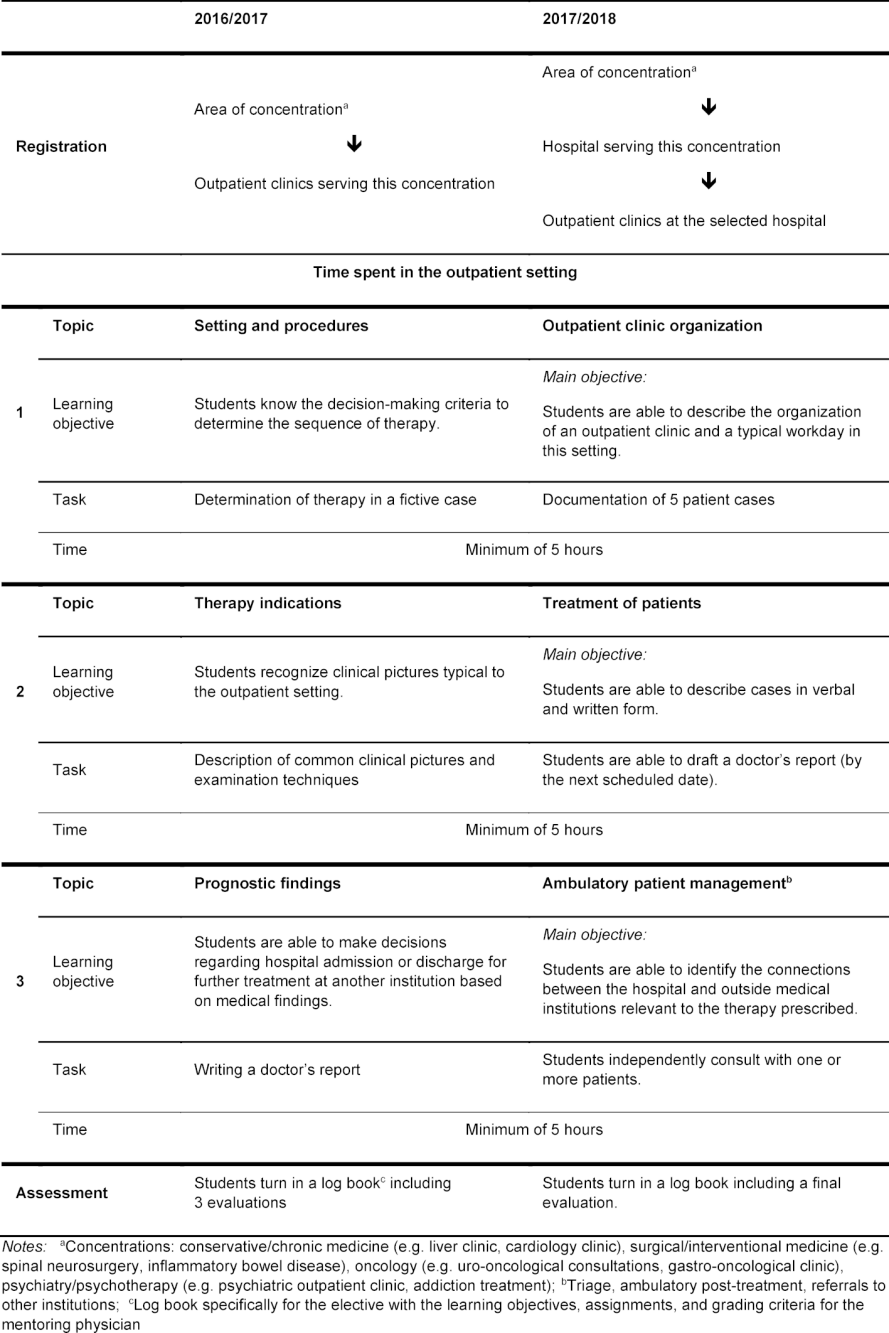
Course sequence for the elective in ambulatory medicine with example learning objectives and assignments from the pilot phase (2016/17) and after revision (2017/18)
